# Known sequence features explain half of all human gene ends

**DOI:** 10.1093/nargab/lqad031

**Published:** 2023-04-05

**Authors:** Aleksei Shkurin, Sara E Pour, Timothy R Hughes

**Affiliations:** Department of Molecular Genetics, University of Toronto, Toronto, ON M5S 1A8, Canada; Terrence Donnelly Centre for Cellular & Biomolecular Research, Toronto, ON M5S 3E1, Canada; Department of Molecular Genetics, University of Toronto, Toronto, ON M5S 1A8, Canada; Terrence Donnelly Centre for Cellular & Biomolecular Research, Toronto, ON M5S 3E1, Canada; Department of Molecular Genetics, University of Toronto, Toronto, ON M5S 1A8, Canada; Terrence Donnelly Centre for Cellular & Biomolecular Research, Toronto, ON M5S 3E1, Canada

## Abstract

Cleavage and polyadenylation (CPA) sites define eukaryotic gene ends. CPA sites are associated with five key sequence recognition elements: the upstream UGUA, the polyadenylation signal (PAS), and U-rich sequences; the CA*/*UA dinucleotide where cleavage occurs; and GU-rich downstream elements (DSEs). Currently, it is not clear whether these sequences are sufficient to delineate CPA sites. Additionally, numerous other sequences and factors have been described, often in the context of promoting alternative CPA sites and preventing cryptic CPA site usage. Here, we dissect the contributions of individual sequence features to CPA using standard discriminative models. We show that models comprised only of the five primary CPA sequence features give highest probability scores to constitutive CPA sites at the ends of coding genes, relative to the entire pre-mRNA sequence, for 59% of all human genes. U1-hybridizing sequences provide a small boost in performance. The addition of all known RBP RNA binding motifs to the model increases this figure to only 61%, suggesting that additional factors beyond the core CPA machinery have a minimal role in delineating real from cryptic sites. To our knowledge, this high effectiveness of established features to predict human gene ends has not previously been documented.

## INTRODUCTION

**Figure 1.**
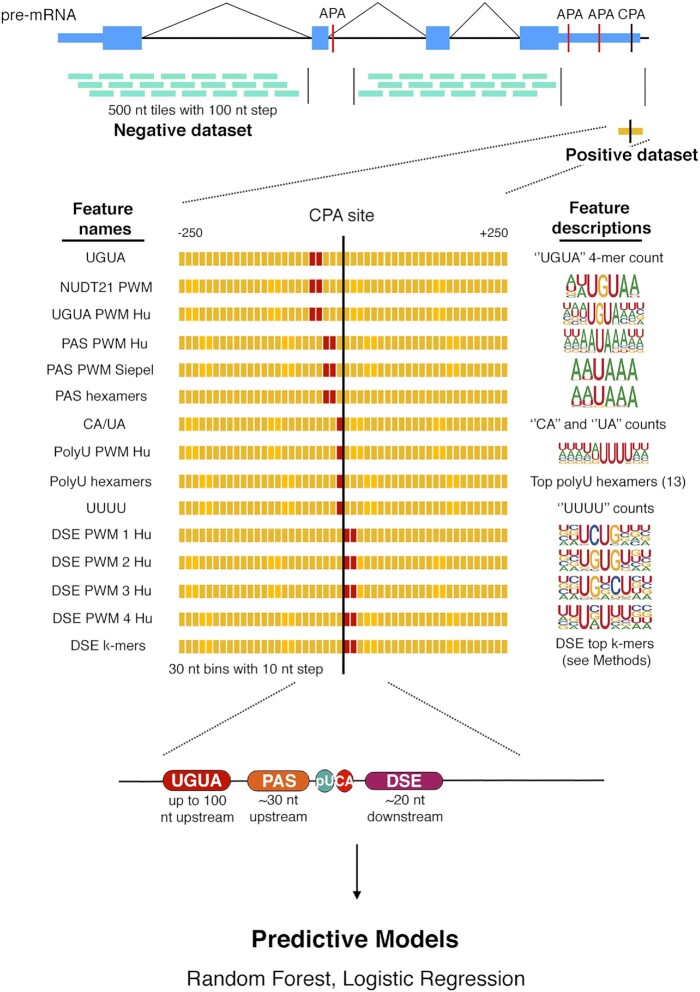
Schematic of the baseline model pipeline. CPA sites from PolyA_DB 3 (37) were processed to identify one constitutive CPA site per gene; 500 nt sequences surrounding these sites are used as the positive dataset. The negative dataset consists of 500 nt portions of genes not overlapping with any annotated CPA sites. The feature matrix is calculated using PWMs scores or k-mer counts within tiling windows. Red positions within the grid indicate the expected positions of each corresponding element, as illustrated below the grid. AUROC and AUPRC are used as evaluation metrics.

Cleavage and polyadenylation (CPA) is the process of cleaving precursor mRNA and adding a string of adenine (A) nucleotides to the 3’-end of a primary RNA transcript (1,2). In humans, CPA is mediated by four main protein complexes (the ‘core’ CPA machinery) that recognize five *cis-*acting RNA elements in the pre-mRNA (3) (Figure 1). First, one or more instances of UGUA are usually found up to 100 nt upstream of the CPA site. The UGUA elements are recognized by NUDT21*/*CFIm25, a subunit of Cleavage Factor Im (CFIm) (2,4,5). Second, the polyadenylation signal (PAS), typically either AAUAAA or AUUAAA (or 11 minor variants), is found around 30 nt upstream the CPA site (6,7). The PAS is the best known of the sequence signals, as it is found in the majority of known human CPA sites (8,9). It is recognized by the CPSF (Cleavage and Polyadenylation Specificity Factor) subunit WDR33, likely in conjunction with CPSF4*/*CPSF30 and CPSF1*/*CPSF160 (10,11).

The endonuclease subunit CPSF3*/*CPSF73 mediates the cleavage, with the cleavage site usually preceded by a CA or UA dinucleotide (2). Poly-U sequences, preferred by CPSF4*/*CPSF30 (12), are also often found surrounding the cleavage site (13), and sometimes further upstream (14). Finally, degenerate U and GU-rich downstream elements (DSEs) are often found starting 20 nucleotides downstream of CPA sites (15). These elements are recognized by CSTF2*/*CstF-64, the RRM-containing subunit of the Cleavage Stimulation Factor complex (16). Consistent with the fundamental importance of CPA, its dysregulation is associated with a wide range of genetic disorders. For example, a mutation within the PAS of FOXP3 (AATAAA to AATGAA) leads to immunodysregulation polyendocrinopathy (17), while mutation of AATAAA to AATACA in TP53 increases susceptibility to cancers including cutaneous basal cell carcinoma, prostate cancer, glioma and colorectal adenoma (18). Similarly, dysregulation of U-rich upstream elements was associated with conditions affecting inflammatory hypercoagulation and tumor invasion (19).

Despite this detailed knowledge, the precise RNA sequence cues that determine actual CPA sites remain a topic of active research. Collectively, an exact match to all of the sequence features above has the potential for relatively high specificity, such that only one or a few sites would be expected in random sequence of the average size of a human pre-mRNA (23 kb) (see Materials and Methods for estimates). However, CPA sites are heterogeneous, with each containing a different assembly of sequence features; combinations of only a subset of the CPA sequence elements would occur much more often. Indeed, ‘cryptic’ CPA sites, which would lead to truncated transcripts, appear to be widely distributed, and at least one mechanism is known to suppress usage of these sites (the U1 snRNP) (20,21).

Metazoans appear to have taken advantage of this flexibility in CPA, with most genes containing multiple CPA sites that produce functional transcript isoforms differing in their terminal exons or 3’ UTR length (3,22,23), thus impacting the protein sequence and*/*or regulation of the transcript. Alternative CPA sites are often tissue-specific (22,24,25) and presumably there are specific mechanisms that dictate their usage. Indeed, there are several examples where corresponding regulators have been identified. For example, the neuronal RBP Nova acts as an inhibitor when binding close to CPA sites, and as an enhancer when binding distant from CPA (26), while neural Hu proteins inhibit CPA sites with U-rich elements (27). A series of previous computational analyses have sought to predict CPA sites from RNA sequence. CPA is a tractable computational problem in which the goal is to find patterns of sequence features that discriminate actual CPA sites from the remainder of the gene. Sequence elements and their positions can be described as a vector that is compatible with probabilistic inference methods.

Early efforts to computationally predict human CPA (28,29) used motif thresholding and quadratic discriminant analysis, respectively, to show that the sequence determinants known at the time (PAS and DSE) have significant classification ability (e.g. 79% accuracy (29)). More recently, CPA site prediction has increasingly used machine learning with larger feature sets encompassing both k-mers and known RNA binding motifs for proteins. Better overall statistical performance has been reported, but the data sets and evaluation criteria employed varied dramatically, complicating direct comparisons among studies. Xie *et al.* (30) used 3-mers derived from top variants of the PAS as input into an HMM-SVM and reported accuracy of 85%. Hafez *et al.* (31) used an SVM trained on 100 nt around CPA sites to obtain an area under the receiver operating curve (AUROC) of 0.996, but employed only the terminal exon sequences as negatives. Leung *et al.* (32) used both ‘hand-crafted’ feature vectors (composed of RBP RNA binding motifs and k-mers) and k-mer sets learned directly from the sequence, employing convolutional neural networks to directly learn alternative polyadenylation patterns, and reported AUROC of 0.97 (hand-crafted) and 0.98 (*k*-mers learned directly) at the task of discriminating CPA sites from neighbouring genomic sequence. None of these papers explicitly report CPA site predictions genome-wide, and do not address why CPA does not occur elsewhere in the primary transcript, which is typically many times longer than the terminal exon. AUROCs in this range would be expected to predict many CPA sites per human gene, on average (AUROC of 0.99 would be roughly equivalent to 1 out of 50 randomly selected sequences scoring as a false positive).

Overall, several critical issues remain unresolved. First, none of the previous studies addressed whether the five well-established sequence features can indeed specify known constitutive CPA sites relative to all non-CPA sequence within primary transcripts. Second, it is difficult to compare the results of previous analyses because different sets of sequences and evaluation metrics were used. Third, linking k-mers to biological mechanisms can be challenging. For example, Hafez *et al.* (31), which used k-mers as features to generate a model with good predictive ability, provided very limited mechanistic explanation (primarily a sequence logo reflecting the general pattern of the most informative sequences relative to CPA, which resemble known regulatory elements). Fourth, until the recent availability of large 3’-end seq datasets, many studies used reference databases that filter out potential CPA sites lacking the established PAS sequence, thus introducing circularity.

Here, we dissect the contributions of diverse RNA sequence features to CPA site discrimination, with the goals of simultaneously increasing performance in a realistic test framework (i.e. with a large excess of negatives derived from real genic sequences) and deriving a set of minimal features that are sufficient to obtain high performance. We find that standard supervised learning approaches (Random Forests and Logistic Regression), employing a small number of established features represented as either classical position weight matrix (PWM) motifs or a handful of short k-mers, are surprisingly effective at identifying constitutive CPA sites at the ends of human genes. Addition of hundreds of diverse sequence features to the model (U1 binding sites, and all known RBP RNA binding motifs) does not improve the model significantly. Thus, while CPA is potentially controlled by many protein factors, the core CPA machinery alone plays a major role in defining human gene structures.

## MATERIALS AND METHODS

### Initial calculation of specificity of known CPA sequence features in random sequence

The probability of observing UGUA within a 100 base window is 0.39 (100*/*4^4^). The probability of observing any of the 13 variants of the PAS in a 20-base window is 0.063 (13*20*/*4^6^), if they are weighted equally, or 0.022, if they are weighted by their probability at CPA sites (as a proxy for activity). The probability of observing UUUU in a 30 base window is 0.11 (30*/*4^4^). The probability of observing a CA or UA dinucleotide is 0.13. The probability of observing G or U for eight consecutive bases (taken as a strong DSE (33) within a 40 base window is roughly estimated as 0.156 (40*/*2^8^). The expected frequency of all five features occurring surrounding any base is 0.39*0.063*0.11*0.13*0.156 = 0.000055, or one every 18 kb if the 13 PAS variants are weighted equally (every 52 kb with the PAS sites weighted). We note that the low G*/*C content of the human genome would make these A*/*U-rich sequences more likely.

### Data sources

Human CPA site annotations were obtained from PolyA DB 3 (34). To select constitutive CPA sites, we developed a significance metric by multiplying percentage of samples expressed (PSE) and mean reads per million (RPM) scores provided by PolyA DB 3. Sites that were selected for positive training dataset are those that have highest PSExRPM score and located in (or at the end of) the 3^’^UTR of the longest isoform of the corresponding UCSC gene on the table browser. We retrieved 250 nt of sequence around each constitutive CPA site from UCSC (hg38).

To create a negative dataset for training the classifiers, we tiled the same longest UCSC isoforms genes into 500 nt windows with 100 nt steps and removed those that overlap any CPA sites in PolyA DB 3. For training, we then randomly subset negative sequences to obtain a 30:1 ratio of negative to positive data.

PWMs used for this study were taken from CISBP-RNA database (35) and ENCORE (36). We derived the ‘Hexamer’ PAS PWM by weighting each hexamer in (8) according to its counts in the constitutive CPA sites described above. DSE PWMs were obtained from (13). To score U1 sites we used the 5’SS MaxEntScan score (37) and RNAhybrid (38). The ‘Siepel PAS’ site is from (39). The ‘DSE *k*-mers’ feature is calculated as the count of all instances of ‘G’, ‘U’, ‘GG’, ‘GU’, ‘UG’ and ‘UU’. The ‘UGUA’ feature is a PWM representing this single sequence.

### Feature matrices

To calculate feature matrices, we break each individual 500 nt sequence from positive and negative data into bins of size 30 nt with a 10 nt step. Next, for each of the PWMs in each 30 nt bin, we calculate the maximum log(odds) score in each of those bins and convert these predicted energy scores to predicted affinity. For MaxEntScan and RNAhybrid, we also select the maximum score per each bin (leaving MaxEntScan in log domain).

### Machine learning

Random Forest and Logistic Regression classifiers were trained in Python (v3.5.1) using scikit-learn library version 0.22.2. Random Forest training used RandomForestClassifier with 30 000 trees, a minimum sample split of 5, class weight ‘balanced’. For the baseline Logistic Regression, we selected ‘l1’ penalty, regularization strength of 0.1, tolerance of 0.01, ‘saga’ solver, and ‘balanced’ class weight. The constitutive vs cryptic Logistic Regression model used the same parameters as before, with the exception of a regularization strength of 0.0018. We tested several regularization parameters and determined that performance sharply decreased after this value.

## RESULTS

### Compilation of CPA data and ‘core’ CPA motifs

We began by organizing a system to computationally interrogate the contributions of the five established *cis-*acting RNA elements, and their positions relative to the cleavage site (Figure 1 shows a schematic). This system is comprised of four basic components: (i) a dataset of CPA sites (positives), and non-CPA sites (negatives); (ii) motif models (i.e. PWMs), individual k-mers, and other scores that represent predicted affinity of RBPs to any given sequence, and scores obtained from these models for tiled sequence windows relative to the CPA sites; (iii) algorithms that input the RNA binding motif scores for each tiling window as features, and output both a probability that reflects confidence that any given example is a CPA site, as well as information about the relative importance of the individual features and (iv) a testing regime, which quantifies the predictive ability of each algorithm using several criteria. Implementing each of these components involves numerous choices. In each case, we sought to minimize bias and circularity, and to achieve results that are mechanistically interpretable, to be biologically meaningful, and as simple as possible, to avoid overfitting and ambiguity.

For the dataset of CPA sites (‘positives’), we employed PolyA DB 3, which is based on 3’-READS (a 3’-end sequencing method) applied to a panel of cell lines and mixed tissue (34). This database includes 58 676 CPA sites, each associated with values including mean RPKM and PSE (Percentage Samples Expressed). Because our initial goal was to characterize contributions of the core machinery to CPA, and the core machinery is presumably constitutive, we selected our initial set of positives as 15 794 CPA sites (allowing only one per UCSC gene) with both high RPKM and PSE scores that overlap or flank 3’UTRs (see Materials and Methods). We refer to these as ‘constitutive’ CPA sites. We excluded all other CPA sites annotated in PolyA DB 3, as they represent alternative CPA sites. We used a 500 nt window to represent each CPA site (–250 to +250). We generated ‘negative’ sequences (i.e. those that are not CPA sites) by first collecting all 500-nt tiling windows (with offset 100 nt) in the sense strand of genes with constitutive CPA sites, and then removing windows that overlap more than 10% with any CPA site in PolyA DB 3. This process generated 15 352 546 negative examples; generally, only a randomly selected subset of 100 000 were used for training the models, and 400 000 employed for testing. We generated training and testing sets by splitting the chromosomes (Chromosomes 1–14 are used for training, and 15–22 and X are used for testing).

We compiled RNA binding motif models from diverse sources (shown in Figure 1; see Materials and Methods for details) to calculate features in the models. We included multiple representations for each component of the core CPA machinery (e.g. several PAS signals have been described, and to our knowledge it is unknown which best reflects binding of CPSF, or whether a single motif is sufficient). It is unclear whether motif models learned from CPA sites accurately represent the full sequence preferences of these proteins; therefore, where possible, we used RNA binding motifs derived from data collected without using knowledge of established CPA sequences (e.g. from *in vitro* assays such as RNAcompete (40)). We note that some of the motifs have been learned from human mRNA sequences present in the training data, which could lead to circularity, but we also note that the motif representations are simple (and thus less likely to be overfitted). To generate a feature vector for each CPA site (positive or negative), we scored windows of length 30 bases, in 10 base tiling steps, over each 500 nt sequence. The motif scores were represented in linear domain (i.e. 10^log(odds)^), which we reasoned would reflect relative preference (i.e. relative Ka). We used the max score for each window. Thus, with 48 windows and 15 different representations of the elements (Figure 1), there are initially 720 features comprising the feature vector for each example sequence.

For the learning algorithms, we employed two commonly used implementations of different machine learning strategies. The first, Random Forests (RF), employs a large set of decision trees, which has the advantage that it inherently captures logic relationships and is thought to be less prone to overfitting because it uses an ensemble of decorrelated classifiers. It can also be used to obtain importance scores for each feature. The second, Logistic Regression (LR) with L1 regularization, does not inherently capture logical relationships, but it has the advantage that the feature selection through Lasso regularization tends to collapse redundant features, giving zero weight to noninformative features. The directional weights (coefficients) given to each feature are easily obtained.

### Performance of prediction methods confirms critical importance of PAS and DSE

**Figure 2.**
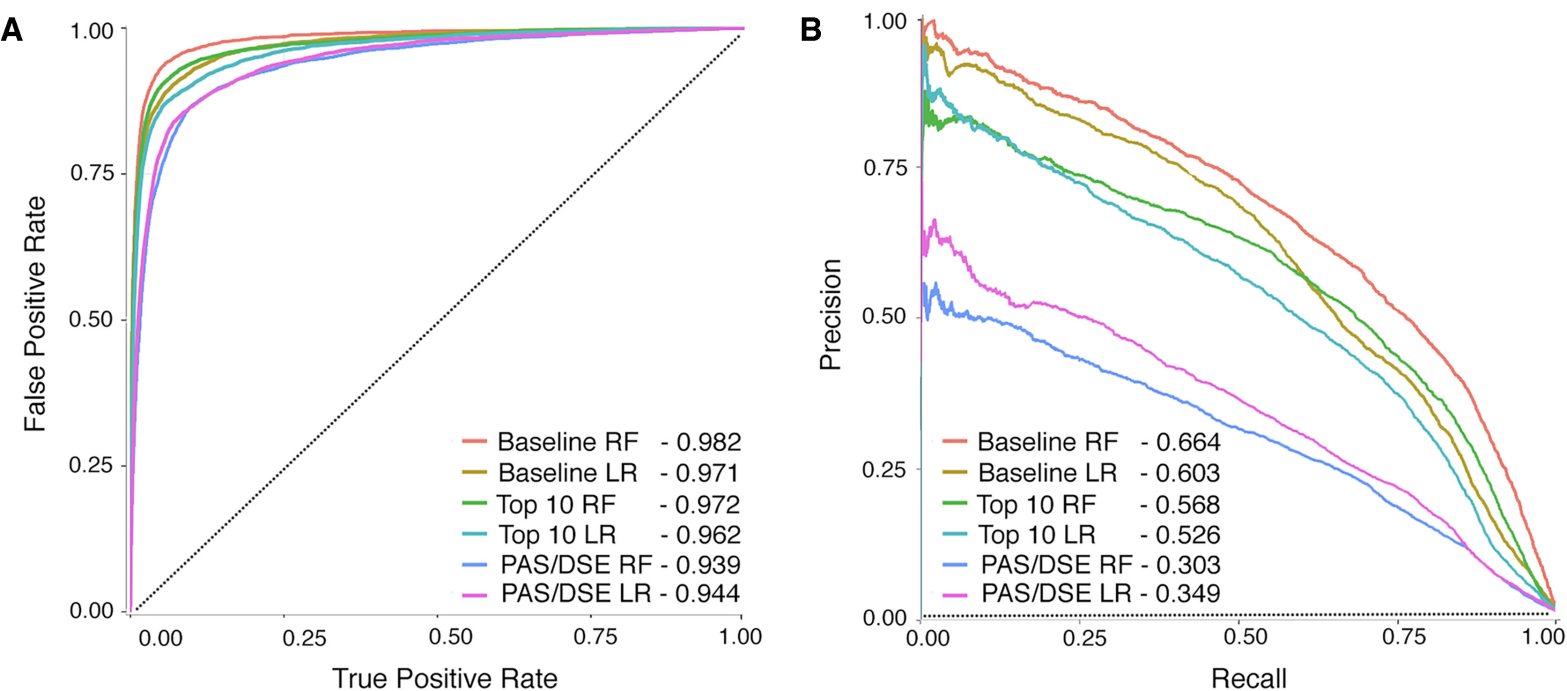
Performance of the baseline models (i.e., those built using elements recognized by known CPA machinery). (**A**) Receiver operating curve (ROC); (**B**) precision recall curve (PRC), with a 50-fold excess of negatives. Models shown on the plots include baseline RF and LR models with all 720 features, Top10 RF and LR models with only Top10 features based on LR weights, and PAS/DSE RF and LR models that are built using only two features (i.e., PAS and DSEs), as described in the text. The legends show area under the curve values for each model. Dotted lines show the performance of a random guessing classifier.

**Figure 3.**
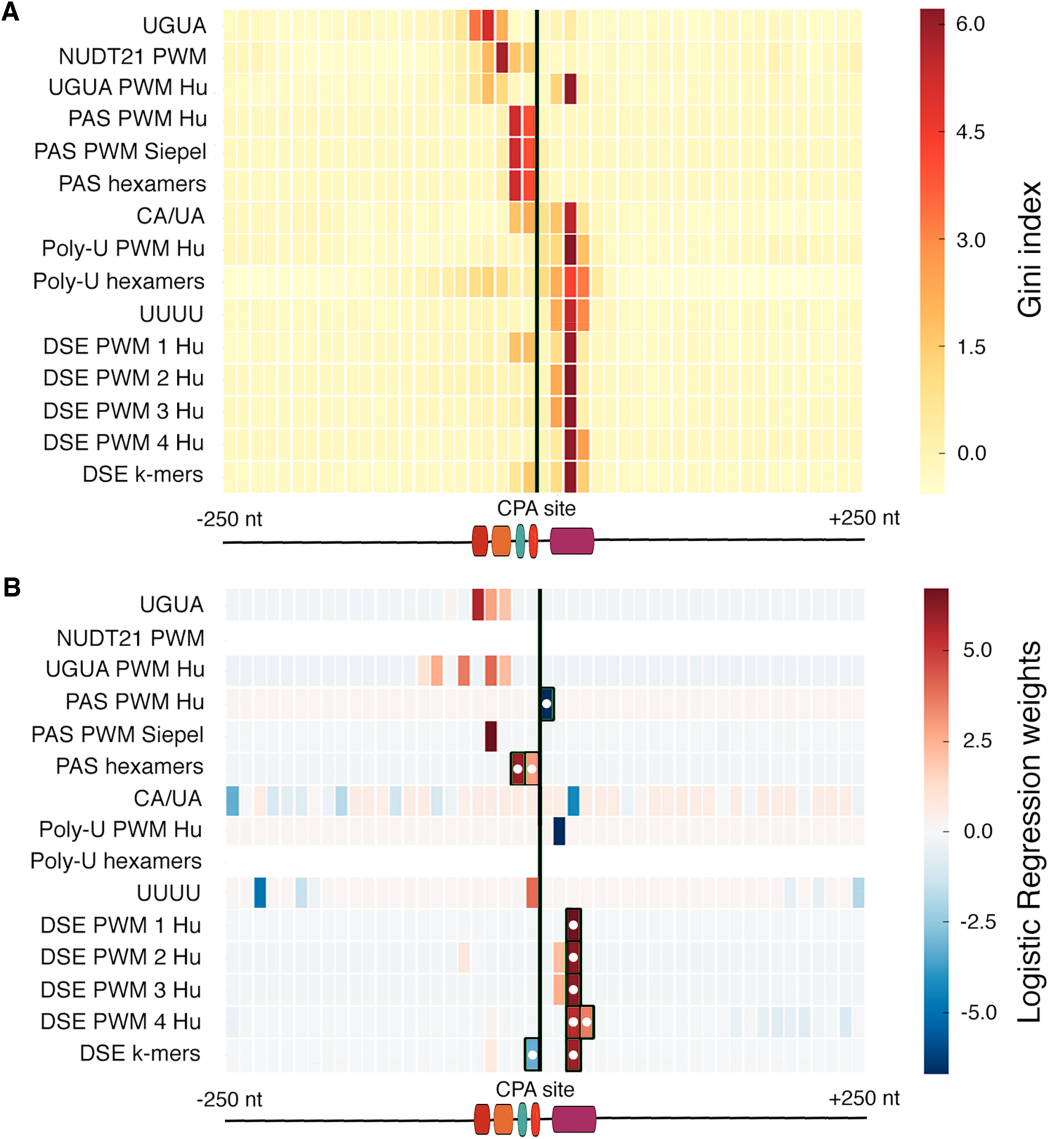
Heatmaps showing feature importance scores identified from (**A**) RF and (**B**) LR baseline models. Black vertical lines indicate the location of the CPA site relative to top-scoring features. In panel (B), the matrix rows are normalized prior to plotting, and black boxes and white dots indicate the Top10 features.

To evaluate the performance of the models, we report both AUROC (Area under the Receiver Operating Curve) and AUPRC (Area under the Precision Recall Curve), with a 30-fold excess of negatives to positives (which represents a large excess but avoids unwieldy run times). Figure 2 shows these values for six different variants of the predictors, which together with the feature importance scores and weights (Figure 3) enable dissection of the contributions of the core machinery. The first two models are the initial RF and LR models, with 720 features per example; we refer to the 720 feature RF model as the ‘baseline’ model. These are the best performing models (Figure 2), and RF and LR are in reasonable agreement regarding which features are important (Figure 3). Strikingly, the AUROC values obtained (0.98 and 0.97, respectively) are similar or better to those reported in previous studies that employed more complex and less interpretable models (30–32). The models largely confirmed the regions where the CPA factors are known to act, with the PAS most critical 20–30 bases upstream of the CPA site, and the DSE at 10–20 bases downstream. The feature importance scores for the models differ somewhat, presumably because only LR involves a regularization step, in which the number of weighted features is minimized, but overall, the two models are consistent. The CA*/*UA dinucleotide appears to be dispensable to both models, perhaps because it occurs frequently at random and controls precise local placement of the CPA site, which was not considered here.

The third and fourth curves (Top10 LR and Top10 RF) in Figure 2 show the performance of RF and LR models encompassing the ten features with highest weights in the baseline LR model (all reside in the PAS and DSE in Figure 3B; indicated with black boxes and white circles). These models are nearly as effective as the full 720 features, albeit with a 10% decrease in AUPRC. Upon further simplification, however, the models are deeply compromised: the fifth and sixth curves in Figure 2, ‘PAS*/*DSE’, are derived using the maximum scores of the three PAS features and seven DSE features within the bins where they are boxed in Figure 3B, respectively. Intriguingly, four very different representations of the DSE all appear to be important, at the same positions: collapsing them into a single value (by taking the maximum score of any of them per bin) results in substantial decline in performance. We speculate that the interaction of CPSF and CstF with each individual sequence and with each other may be more complex than what can be captured by PWM motif models (see Discussion).

### Model predictions on complete pre-mRNAs and pathogenic mutations

**Figure 4.**
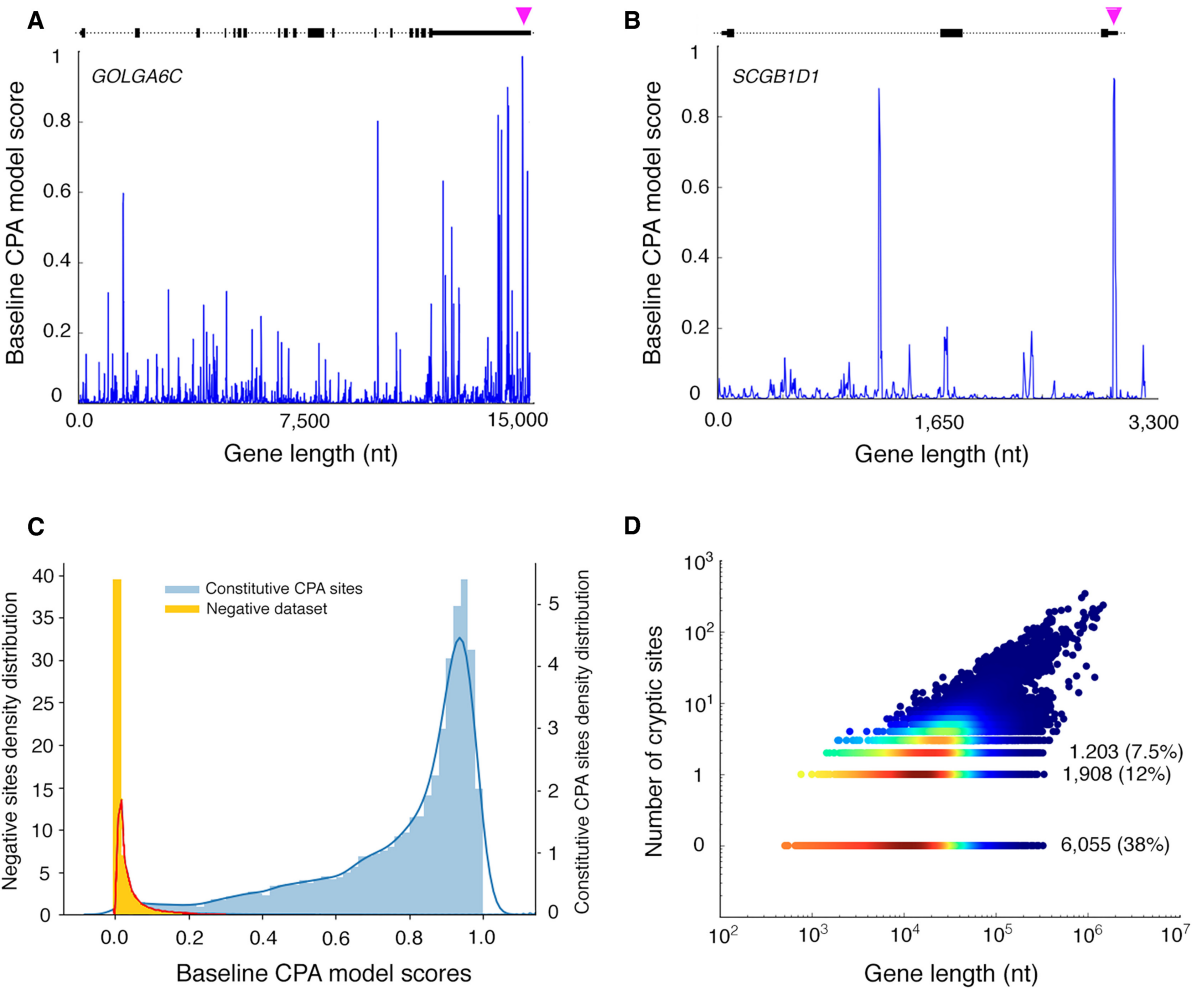
Overview of cryptic CPA sites. (**A**, **B**) Baseline model CPA probability scores for the genes GOLGA6C (A) and SCGB1D1 (B) at base-level resolution. (**C**) Distribution of CPA site probabilities the model assigns to constitutive CPA sites and all other sites. (**D**) Scatter plot showing the number of cryptic sites per gene (i.e. those with *D* > 0.69, grouping adjacent bases exceeding this metric) versus the length of the corresponding gene. The number of genes having 0, 1 and 2 cryptic sites and their proportion are indicated.

We next asked how well the baseline model specifically predicts the 3^’^ ends of genes. To do this, we obtained the primary transcripts from UCSC for the 15 794 human protein-coding genes that had a ‘constitutive’ CPA site, as defined above, and analyzed all possible 500-base tiling windows within the pre-mRNA, i.e. starting at every base. Examples are shown in Figure 4A and B, illustrating that there is little bias in position within the gene; the probability distributions for the constitutive CPA sites and all negative sequences are shown in Figure 4C. Strikingly, the baseline model assigned the highest probability to the ‘constitutive’ CPA site for 59% of all genes. The significance of this outcome is discussed below (see Discussion), but we believe it is a higher figure than would have been anticipated, and we take it to confirm that the core CPA machinery plays a major role in determining not only CPA sites but also gene ends. As expected, short genes are more likely to have the highest scoring sequence at the end of the gene, because the probability of encountering a cryptic site at random would be lower (Figure 4D).

To assess the ability of the baseline model to forecast the consequences of mutation events, we used FOXP3 and TP53 CPA sequences and compared the prediction scores with and without known PAS mutations (17,18). For FOXP3, the original CPA site was assigned a probability of 0.67, and the mutated sequence 0.04. In case of TP53, the performance changed from 0.73 to 0.34. These results show that the model is sensitive to known pathogenic mutations and further demonstrate its ability to identify functional CPA sites.

### Predicting modifiers of CPA beyond the baseline model

**Figure 5.**
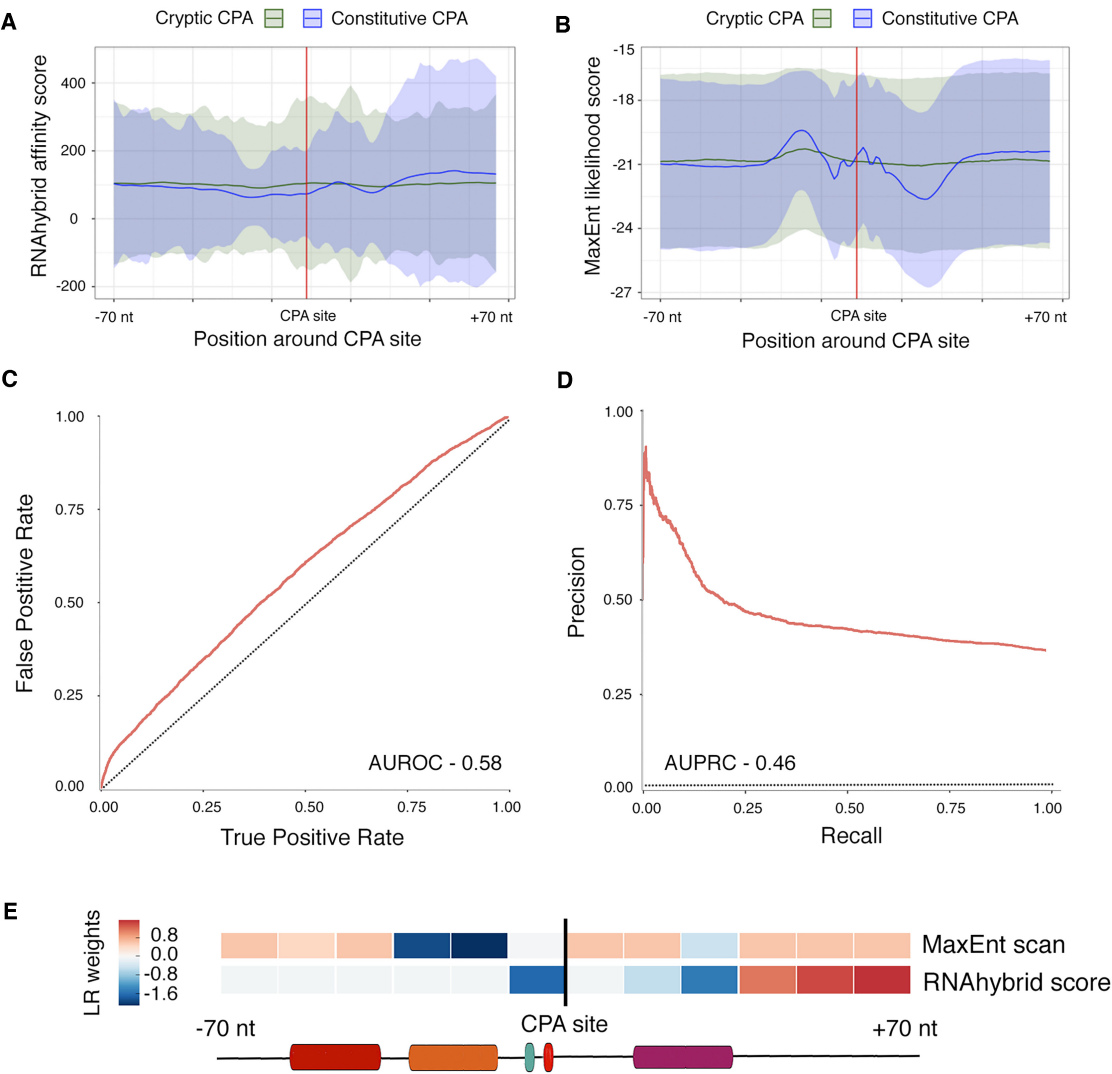
Overview of U1 analyses. (**A**, **B**) Average scores for RNAhybrid (A) and MaxEntScan (B) in a 140 nt sequence window centered on CPA sites. The line shows median and shading shows the standard deviation at each position. (**C**, **D**) ROC and PRC of the cryptic CPA versus constitutive CPA model trained using U1 RNAhybrid and MaxEntScan scores. (**E**) LR weights for RNAhybrid and MaxEntScan scores at each position.

We next asked whether inclusion of additional features in the model would aid in discriminating constitutive from cryptic CPA sites. We defined cryptic sites as those that the baseline model assigned probability scores higher than the overall average for constitutive CPA sites (*D >* 0.69), and that did not overlap with any known CPA sites in PolyA DB 3, therefore excluding the possibility that these sequences are alternative CPA sites. Among the 15 794 genes, there were 40 537 such sites. We formulated the problem as a discrimination task between the constitutive CPA sites and the cryptic sites, instead of discrimination between constitutive CPA sites and randomly selected pre-mRNA sequences as previously, but otherwise applied a similar framework as above. We first examined the ability of U1 recognition elements to discriminate globally between the constitutive and cryptic CPA sites, because U1 can suppress CPA (20,21). We considered two representations of U1 binding: RNAhybrid (38), to calculate binding affinity of the classical U1 7-mer (41) to any given 7 base RNA sequence, and 5’MaxEntScan, which calculates the likelihood that a given 9-mer is a 5’ splice site (of which U1 recognition is a major component) (37). Both models displayed some bias in constitutive versus cryptic CPA sites (lines show medians at each base relative to the predicted CPA site in Figure 5A and B). We observed a slight overall decrease in high-affinity U1 RNAhybrid scores for constitutive CPA sites, as expected if U1 suppresses CPA. MaxEntScan displayed more striking biases, including a depletion following constitutive CPA sites. But, both U1 measures also displayed high standard deviation (shading in Figure 5A and B), and we note that their overall scores would be biased by deviations in base content caused by the core CPA sequences.

We then asked how well the U1 scores serve in a classification framework. Here, we used a sequence window of –70 to + 70, with 20 nt windows tiled every 10 nt, because initial trials with different window sizes indicated that most of the predictive signal is near the center (i.e. the CPA site), and also in order to accommodate a larger number of features (see below). We used an LR model as it allowed us to perform L1 regularization with the goal of reducing potential redundancy in training data and generating a subset of top scoring features. Together, the two U1 representations do provide some classification ability (AUROC of 0.58) (Figure 5C and D), with depletion just upstream of CPA having greatest impact (Figure 5E). By the conventional interpretation of AUROC, addition of these U1 measures represents a 16% performance increase over random guessing.

**Figure 6.**
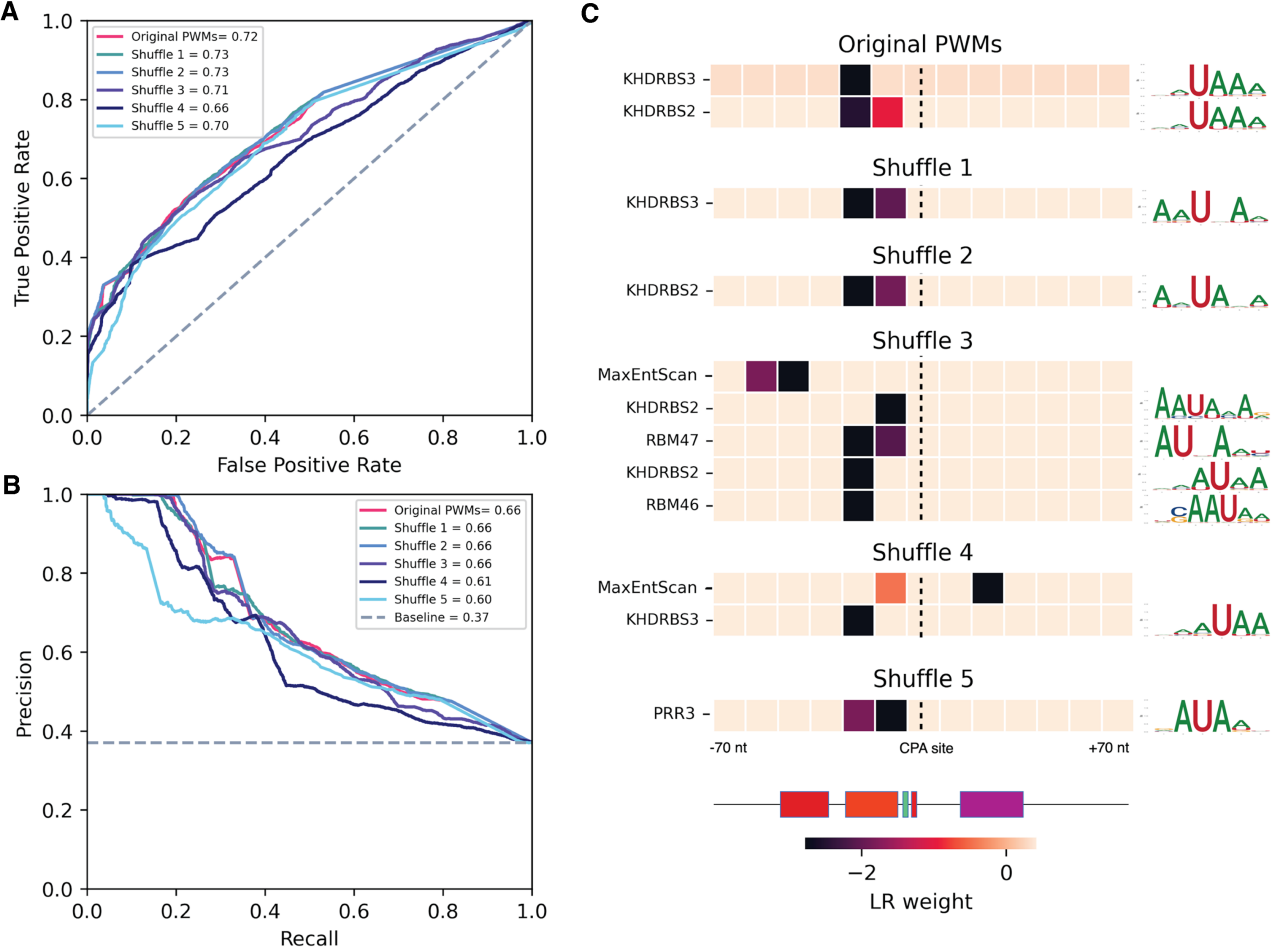
Overview of cryptic versus constitutive CPA model, incorporating all known RBP motifs. (**A**, **B**) ROC and PRC of the model that uses both U1 representations and RBP PWMs, versus models trained using five different shuffles of the same PWMs. (**C**) Heatmaps depicting LR weights for the top scoring RBP PWMs and U1 features, for the original PWMs and each shuffle.

We then extended the constitutive versus cryptic CPA model to include all known RNA binding motifs for human RBPs. Because the number of CPA sites is large, there is sufficient statistical power to simultaneously consider all 324 different PWMs for human RBPs (obtained from CisBP- RNA (35) and from (36)) as well as U1. The LR model resulting from a standard procedure of feature reduction (see Materials and Methods) strikingly retained only two motifs – those of KHDRBS2 and KHDRBS3, both of which closely resemble the PAS and are important only in the same location as the PAS, suggesting that the model mainly detects small variations of the PAS, and that these are important in CPA site recognition (Figure 6). In addition, shuffling of the PWMs in the cryptic CPA model does not result in a drastic decrease in performance. This observation appears to be explained by the fact that shuffling of the KHDRBS2/3 motifs, as well as other motifs, easily produces sequences resembling the canonical PAS (Figure 6), an outcome not surprising given the simplicity of the PAS consensus (AAUAAA).

Finally, we asked whether combining the two models (the ‘baseline’, and the ‘constitutive CPA versus cryptic’ models), by simply multiplying their probabilities, would increase the proportion of genes for which the constitutive CPA site is given the highest score. Indeed, this combined score yielded the highest value at the constitutive CPA site for 61% of all genes (relative to 59% for the baseline model alone). Thus, while a slight increase in specificity is achieved by the two-stage model, there is still a large proportion of gene ends that are not fully explained even by the combined model (discussed below).

## DISCUSSION

A major outcome of this study is that a model utilizing representations of the sequence preferences of only four different components of the ‘core’ CPA machinery has good ability at the difficult task of pinpointing the ends of human genes. To our knowledge, this is the first such global demonstration encompassing the majority of human gene sequences. The model identifies ‘cryptic’ sites, but they are not nearly as prevalent as would be expected from considering only the PAS, which is often used to identify cryptic sites. We propose this outcome to signify that the use of PWMs, and models that can incorporate spatial preferences and (potentially) interactions among the features is beneficial, relative to simple calculations based on the appearance of k-mers and consensus sequences. As a corollary, this outcome also shows that components of the known CPA machinery are quite specific in combination: specificity of the models is cut in half when only simplified PAS and DSE are included.

The models themselves have properties that may reveal intriguing biology. UGUA, for instance, is important to the models only between 30 and 50 nt upstream, although it is described in the literature as appearing *<*100 (13), and the LR model assigns weights up to 100 bp from the CPA site. We note that CFIm is a dimer, such that a second UGUA upstream at a constrained distance may contribute to binding. But, if this were the case, the RF model should have detected it. If the second site did not have a constrained position, it would not be expected to greatly impact specificity. We also note that both the RF and LR models indicate that incorporation of multiple representations of the DSE is important. A trivial explanation would be that the current PWMs are inadequate, but it is also possible that a single PWM motif cannot fully capture the sequence specificity of the RNA binding activity, and*/*or that the DSE sequence impacts the spatial organization of the larger complexes. If so, this observation may provide an explanation why no simple regular expression has emerged that accurately identifies CPA sites. The outstanding question remains as to how bona fide CPA sites are distinguished from cryptic sites.

In our cryptic versus constitutive framework (Figure 6), performance is driven entirely by motifs that resemble the PAS (KHDRBS2/3 in the real motifs, and others in the shuffled motifs). One possible explanation is that the PAS models we utilized are sensitive to subtle variations, and can be improved. We also note, however, that KHDRBS2 is a known regulator of CPA, through its ability to overlap and mask cryptic internal PASs, thereby inhibiting their recognition by the CPA machinery during pre-mRNA processing (42). We also emphasize that the current set of RBP motifs is not comprehensive: there may be missing information, such as an unknown or incorrect motif for a known RBP, or the existence of as-yet unknown RBPs or other factors (e.g. miRNAs or specific secondary structures) that could have affected the model's performance.

It is likely that other aspects of mRNA transcription and processing play a role in definition of gene ends, e.g. by licensing CPA activity. Indeed, the specificity of CPA for RNA polymerase II (Pol II) transcripts is believed to be controlled largely by the physical association of CPA-related proteins with the carboxyterminal domain (CTD) of the Pol II large subunit (43), which is in turn associated with the phosphorylation of Ser2 residues of the CTD heptad repeats (44,45), observed mainly near gene ends (46). Nuclear export factors also accumulate on the terminal exon, and can influence CPA (47). Perturbation of other aspects of transcription and RNA processing (e.g. mRNA capping) can influence polyadenylation (48), suggesting that longer-distance interactions along transcripts can occur.

How all these events are dictated by the DNA and RNA sequence is unclear. The terminal exon structure itself is an obvious candidate: terminal exons are distinguished by large size, and by lacking a 5^’^ splice site. Surprisingly, however, 48% of terminal exons in our data set do contain at least one sequence scoring above 7.5 in MaxEntScan (the median score of bona fide 5^’^ sites we examined in known internal exons); thus, absence of a 5^’^ splice site sequence appears unlikely to be a discriminating factor. It has long been observed that the 3’-terminal intron is important for efficient RNA 3^’^-end formation (49), and *in vitro*, the terminal 3’ splice cite and the CPA site are coupled and mutually reinforcing (50). We speculate that sequence features analogous to splicing enhancers may exist. Identifying such elements is complicated due to the large size of terminal exons and the presence of multiple constraints: both coding sequence and 3^’^ UTRs are often highly conserved, presumably due to mechanism other than CPA specification. Nonetheless, dissecting how other sequence properties of gene ends interact with the core CPA sequence elements, potentially over long distances, may be the key to a complete understanding of how gene ends are recognized. The models we present provide a strong framing for the problem and will also be instrumental in this endeavour.

## Data Availability

We employed public data sources as described above. The constitutive CPA sites, negative samples, matrices for producing heatmaps used in this paper, and all PWMs are posted at https://hugheslab.ccbr.utoronto.ca/supplementary-data/HumanGeneEnds/. Executable code and descriptions thereof are found at https://github.com/AlekseiShkurin/HumanGeneEnds and https://github.com/spour/updated_HumanGeneEnds. Code and data to reproduce results and figures is available at https://doi.org/10.5281/zenodo.7613406.
